# IL7 signaling confers polyfunctionality to antitumor CD4+ T cells

**DOI:** 10.1186/2051-1426-2-S3-P43

**Published:** 2014-11-06

**Authors:** Gang Zhou, Zhi-Chun Ding

**Affiliations:** 1 Georgia Regents University, Augusta, GA, United States

## 

Mounting evidence indicates that polyfunctional T cells, effector T cells capable of simultaneously producing multiple pro-inflammatory cytokines, are more efficacious in controlling infection and cancer. Our previous study reported that adoptive transfer of tumor-specific CD4+ T cells following chemotherapy gave rise to polyfunctional CD4+ effector cells, characterized by concomitant expression of CD40L, IFNγ, TNFα, IL2 and granzyme B. One unique feature of these highly activated polyfunctional CD4+ effector cells was the high level expression of IL7 receptor, suggesting that IL7 plays a critical role in the generation and maintenance of these cells. In this project, we studied how IL7 signaling confers polyfunctionality to CD4+ T cells. We found that only IL7, but not other IL2 family cytokines can promote the acquisition of polyfunctionality in naïve CD4+ T cells upon antigenic stimulation in vitro. IL7 signaling resulted in increased histone acetylation in the promoters of effector molecules including IFNgamma and IL2. Mechanistically, PI3K activation was required for polyfunctionality. Administration of rIL7 following chemotherapy and CD4+ T cell adoptive therapy led to enhanced and sustained presence of polyfunctional CD4+ effector cells which mediated durable antitumor effects in mice with advanced B cell lymphomas. Our results provide novel insights into the mechanisms by which IL7 drives the generation of polyfunctional CD4+ effector cells.

